# High Light Intensity Leads to Increased Peroxule-Mitochondria Interactions in Plants

**DOI:** 10.3389/fcell.2016.00006

**Published:** 2016-02-04

**Authors:** Erica-Ashley Jaipargas, Neeta Mathur, Firas Bou Daher, Geoffrey O. Wasteneys, Jaideep Mathur

**Affiliations:** ^1^Laboratory of Plant Development and Interactions, Department of Molecular and Cellular Biology, University of GuelphGuelph, ON, Canada; ^2^Department of Botany, The University of British ColumbiaVancouver, BC, Canada

**Keywords:** peroxisomes, peroxules, mitochondria, organelle interactions, *any1* mutant, ER

## Abstract

Peroxules are thin protrusions from spherical peroxisomes produced under low levels of reactive oxygen species (ROS) stress. Whereas, stress mitigation favors peroxule retraction, prolongation of the ROS stress leads to the elongation of the peroxisome into a tubular form. Subsequently, the elongated form becomes constricted through the binding of proteins such as dynamin related proteins 3A and 3B and eventually undergoes fission to increase the peroxisomal population within a cell. The events that occur in the short time window between peroxule initiation and the tubulation of the entire peroxisome have not been observed in living plant cells. Here, using fluorescent protein aided live-imaging, we show that peroxules are formed after only 4 min of high light (HL) irradiation during which there is a perceptible increase in the cytosolic levels of hydrogen peroxide. Using a stable, double transgenic line of *Arabidopsis thaliana* expressing a peroxisome targeted YFP and a mitochondrial targeted GFP probe, we observed sustained interactions between peroxules and small, spherical mitochondria. Further, it was observed that the frequency of HL-induced interactions between peroxules and mitochondria increased in the Arabidopsis *anisotropy1* mutant that has reduced cell wall crystallinity and where we show accumulation of higher H_2_O_2_ levels than wild type plants. Our observations suggest a testable model whereby peroxules act as interaction platforms for ROS-distressed mitochondria that may release membrane proteins and fission factors. These proteins might thus become easily available to peroxisomes and facilitate their proliferation for enhancing the ROS-combating capability of a plant cell.

## Introduction

**Figure 1:**
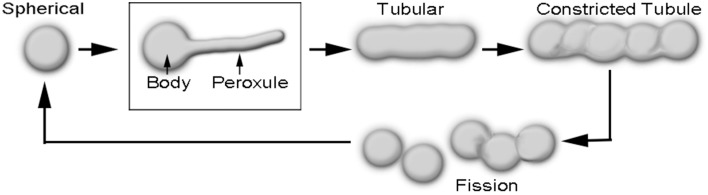
**Diagrammatic depiction of sequential changes in peroxisome morphology leading to their fission and proliferation**. Peroxules extend and retract from a spherical peroxisome body and thus represent a transient, intermediate state between the spherical and the completely tubular, pre-constriction form of peroxisomes.

Peroxisomes are directly implicated in the scavenging of reactive oxygen and reactive nitrogen species (ROS and RNS, respectively) in plant cells (Corpas et al., 2013; Corpas, 2015). Peroxisome morphology in plants varies from ca. 0.5 to 2 μm diameter spheres to ca. 3–8 μm long tubules. Whereas, the spherical shape is typical for peroxisomes in a cell that is not overtly stressed, the elongated form is usually observed in a stressed cell and indicates that the peroxisome will soon undergo fission to produce more spherical peroxisomes (Figure 1; Schrader, 2006; Sinclair et al., 2009; Delille et al., 2010; Barton et al., 2014; Schrader et al., 2014). In addition, a transient form is encountered in response to mild ROS stress during which the peroxisome is neither completely spherical nor tubular (Sinclair et al., 2009; Barton et al., 2013). During this phase thin, dynamic protrusions that have been named peroxules are observed extending and retracting from the spherical peroxisome body (Figure 1; Scott et al., 2007; Sinclair et al., 2009). Whereas, mitigation of a transient ROS stress results in peroxule retraction and reversion to the spherical peroxisome stage, prolonged stress, or higher stress intensity leads to the completely tubular peroxisomal form (Sinclair et al., 2009).

The formation of tubular peroxisomes is considered to be a multistep process involving the insertion of peroxisomal membrane proteins (PMPs) into the existing peroxisomal membrane (Li and Gould, 2003; Koch et al., 2004; Thoms and Erdmann, 2005; Schrader, 2006). The role of Peroxin11 (PEX11) isoforms appears to be especially important during the early tubulation phase and the remodeling of the peroxisome membrane (Lingard and Trelease, 2006; Kobayashi et al., 2007; Nito et al., 2007; Orth et al., 2007; Lingard et al., 2008; Delille et al., 2010; Koch et al., 2010). Interestingly, the ectopic expression of PEX11 family proteins from yeast, plants and mammalian systems results in the formation of juxtaposed elongated peroxisomes (JEPs; Koch et al., 2010), and tubular peroxisomal accumulations (TPAs; Delille et al., 2010). Diagrammatic depictions of single peroxisomes in these clusters suggest a strong morphological resemblance to peroxules. Although JEPs and TPAs inform about the role of PEX11 proteins in peroxisome proliferation they have been usually observed in response to overexpression of specific PEX11 proteins over several hours (Delille et al., 2010; Koch et al., 2010). By contrast, peroxules are produced within seconds in plant cells as a normal peroxisomal response to ROS (Sinclair et al., 2009). Nevertheless, the incorporation of additional PMPs (Delille et al., 2010) might be considered as a general mechanism leading to peroxule formation. The idea appears feasible since peroxule formation occurs as an intermediate stage that leads into the formation of tubular peroxisomes (Figure 1). The crucial question that remains unanswered is how the various PMPs and subsequently required fission factors become available so quickly in response to ROS.

Interestingly some of the major components of the peroxisomal division machinery are shared with mitochondria. Both organelles, once they have reached a certain degree of tubulation become constricted through the mechano-chemical activity of the GTPases Dynamin Related Protein (DRP3A and B/ADL2a and b, respectively, in plants: Arimura and Tsutsumi, 2002; Arimura et al., 2004; Logan et al., 2004; Mano et al., 2004; Zhang and Hu, 2009; Dlp1 in mammals: Pitts et al., 1999; Dnm1 in yeast: Bleazard et al., 1999). The GTPases are recruited from the cytosol and anchored to the membrane by FISSION1, a tail-anchored protein localized to both the peroxisome and outer mitochondrial membrane (Fis1/BIGYIN in plants: Scott et al., 2006; Zhang and Hu, 2008; hFis1 in mammals: Yoon et al., 2003; Stojanovski et al., 2004; Koch et al., 2005; Fis1p in yeast: Mozdy et al., 2000; Tieu and Nunnari, 2000). In both organelles membrane constriction produces a beaded appearance that ultimately leads to their fission and consequent increase in the mitochondrial and peroxisomal population within the cell (Barton et al., 2014; Jaipargas et al., 2015). While many details pertaining to the division machinery common to peroxisomes and mitochondria have been worked out it is still unclear whether the relevant proteins are recruited simultaneously to both organelles or becomes available in a hierarchical manner.

In a similar context while strong biochemical links exist between peroxisomes and mitochondria and both organelles respond to very similar stimuli, including ROS (Hoefnagel et al., 1998; Foyer and Noctor, 2003; Apel and Hirt, 2004; Brookes et al., 2004; Schrader, 2006; Schrader and Yoon, 2007; Schumann and Subramani, 2008; Gechev et al., 2010; Bhattacharjee, 2011), their actual behavior and cooperation in a living plant cell has not been visualized. Scenarios that evoke inter-organelle cooperation during ROS stress suggest that metabolites and proteins may be transferred between the two organelles through mitochondrial membrane extensions (Schumann and Subramani, 2008). We postulated that an opposite scenario might also operate whereby peroxisome extensions in the form of peroxules might be involved in interactions with mitochondria.

Here, we have investigated this idea to uncover the role of peroxules during a possible peroxisome-mitochondria interaction. We have used simultaneous imaging of mitochondria and peroxisomes in stable double transgenic lines expressing different fluorescent proteins targeted to the two organelles. Peroxules were induced by exposing wild type Arabidopsis plants to short periods of high light (HL). Further, an Arabidopsis mutant *anisotropy1* (*any1*; Fujita et al., 2013) was found to exhibit increased frequency of peroxules. Our observations clearly show that small, but not elongated, mitochondria cluster around peroxules in sustained interactions. Our work suggests that the plant cell's ability to combat increased subcellular ROS levels relies upon a hierarchical relationship between mitochondria and peroxisomes where peroxules act as extended platforms for mitochondrial interactions.

## Materials and methods

### Double transgenic plants for simultaneous visualization of peroxisomes and mitochondria

Peroxisomes and peroxules were visualized in a transgenic Arabidopsis line expressing a yellow fluorescent protein (YFP) with a peroxisome targeting tri-peptide (SKL) appended to the carboxy terminus (YFP-PTS1; Mathur et al., 2002). Mitochondria were observed using a transgenic line expressing the N-terminal pre-sequence of the mitochondrial β-ATPase subunit fused to GFP (mitoGFP; Logan and Leaver, 2000). The two lines were crossed to obtain a double transgenic line where peroxisomes and mitochondria could be visualized simultaneously. YFP-PTS1 and mitoGFP were introduced into the *any1* mutant background by crossing with the respective wild type lines. The *any1* phenotype of isotropic expanded hypocotyl cells and trichomes as well as the GFP and YFP fluorescence were confirmed through light and epi-fluorescent microscopy. Double transgenic lines of *any1*-mitoGFP X YFP-PTS1 were created by crossing and stabilized over three generations.

An ER lumen-retained red fluorescent protein probe (RFP-ER; Sinclair et al., 2009) was introduced into stable mitoGFP YFP-PTS1 plants using the *Agrobacterium* floral dip method (Clough and Bent, 1998) (YFP-PTS1 mitoGFP RFP-ER). These triple transgenics were used to further investigate the rearrangements of the ER in response to HL and its role in the clustering of peroxisomes, mitochondria, and chloroplasts.

Seeds were germinated on Murashige and Skoog's medium (Murashige and Skoog, 1962) containing Gamborg B5 vitamins (M404; PhytoTechnology labs) and 3 g/L of Phytagel (Sigma-Aldrich), supplemented with 3% sucrose and with a pre-autoclaving pH adjusted to 5.8. All seeds were stratified for 2 days at 4°C.

### Microscopy

Simultaneous imaging of GFP, YFP, RFP, and chlorophyll was carried out on a three channel Leica TCS-SP5 confocal laser-scanning unit equipped with 488 nm Ar and 543 nm He-Ne lasers. Emission spectra acquired were: GFP—503 to 515 nm (green); RFP- 540 to 630 nm (red); chlorophyll—650 to 710 nm (false colored blue). In double and triple transgenic plants the YFP fluorescence was picked up in both GFP and RFP channels and appeared yellow in the merged images.

Arabidopsis transgenics expressing cytosolic HyPer, a H_2_O_2_ responsive probe (http://www.evrogen.com/products/HyPer/HyPer.shtml, Evrogen, Russia; Belousov et al., 2006; Costa et al., 2010) were used to observe H_2_O_2_ formation in response to a short HL stimulus. Seedlings were given 1–5 min of HL and the change in fluorescence (ex. 488 nm; Em. Band collection 530–560 nm) was analyzed immediately after. The ImageJ RGB Profile Plot plugin was used to determine the changes in fluorescence intensity before and after HL treatment.

All images were captured at a color depth of 24 bit RGB. Tissue and 7–10 day old seedlings were mounted in tap water on a glass depression slide and placed under a coverslip. All images and movies were cropped and processed for brightness/contrast as complete montages or image stacks using either Adobe Photoshop CS3 (http://www.adobe.com) or the ImageJ/Fiji platform (http://fiji.sc/Fiji). Adobe Photoshop was used for annotation of movies.

### Assessing light-induced stress

#### Induction of HL stress

Plants were all grown at low to intermediate light (50–164 μmol m^−2^ s^−1^). Respective controls of the effects of light involved growing plants in complete darkness from stratification up to and including observations, unless stated otherwise. To evaluate the responses and the rapidity of the responses of the organelles to HL, plants were given short exposures (1–5 min) of HL (850 ± 50 μmol m^−2^ s^−1^). Observations were taken immediately after the light treatments to give a better sense of how quickly responses may be.

#### 3,3′-diaminobenzidine staining

To look at the effects of light-induced production of H_2_O_2_, Columbia and *any1* plants were grown in the light (164 μmol m^−2^ s^−1^) for 8 days, transferred to the dark for 24 h and then exposed to light (164 μmol m^−2^ s^−1^) for 30 min, 1 h, or 2 h. Cotyledon and hypocotyl tissue were submerged in a solution of 3,3′-diaminbenzidine (DAB) with a metal enhancer (SIGMA*FAST*™ DAB with Metal Enhancer, Sigma-Aldrich) or distilled water (control) and left under vacuum (−50 KPa) for 4 h. The tissue was cleared with ethanol by washing the samples with 100% ethanol, incubating them in 85% ethanol + 15% methanol overnight, and rinsing them in 70% ethanol and then distilled water. The samples were mounted in 50% glycerol and sealed. All images were acquired at the same light intensity and microscope settings to permit direct comparisons between treatments. The DAB stain intensity was measured as the average inverse gray value using ImageJ (http://imagej.nih.gov/ij/), which was subtracted from the background and considered as the average inverse gray values of distilled water treated seedlings. The staining intensity was representative of the amount of H_2_O_2_ produced during the relative light intensity treatments.

#### Characterizing *anisotropy1* mutant in relation to wild type

Scanning electron microscopy (SEM) and toluidine blue O (TBO) staining were used to assess the consequences of alteration of the cell wall in the *anisotropy1* (*any1*) mutant and the extent of its anisotropy. Scanning electron microscopy (SEM) of Arabidopsis wild type (ecotype Columbia) and *any1* plants grown in soil under 125 ± 10 μmol m^−2^ s^−1^ light intensity was carried out using uncoated tissue in a Tabletop Hitachi TM-1000 microscope with an electron beam accelerated at 15 kV. Leaf cross sections of 12 day old seedlings grown in low light (70 μmol m^−2^ s^−1^) were stained with TBO to analyse the alterations in cell isotropy and cell-to-cell connectivity.

### Statistical analysis

All experiments were carried out at least five times. Observations of mitochondrial length comparisons and their interactions with peroxisomes and peroxules were made with a minimum sample size of *n* = 50. Two-tailed *t*-tests were made to determine the significance of results. Significance was predetermined as having a *p* < 0.01 (99% confidence interval).

## Results

### HL irradiation increases cytosolic H_2_O_2_

**Figure 2:**
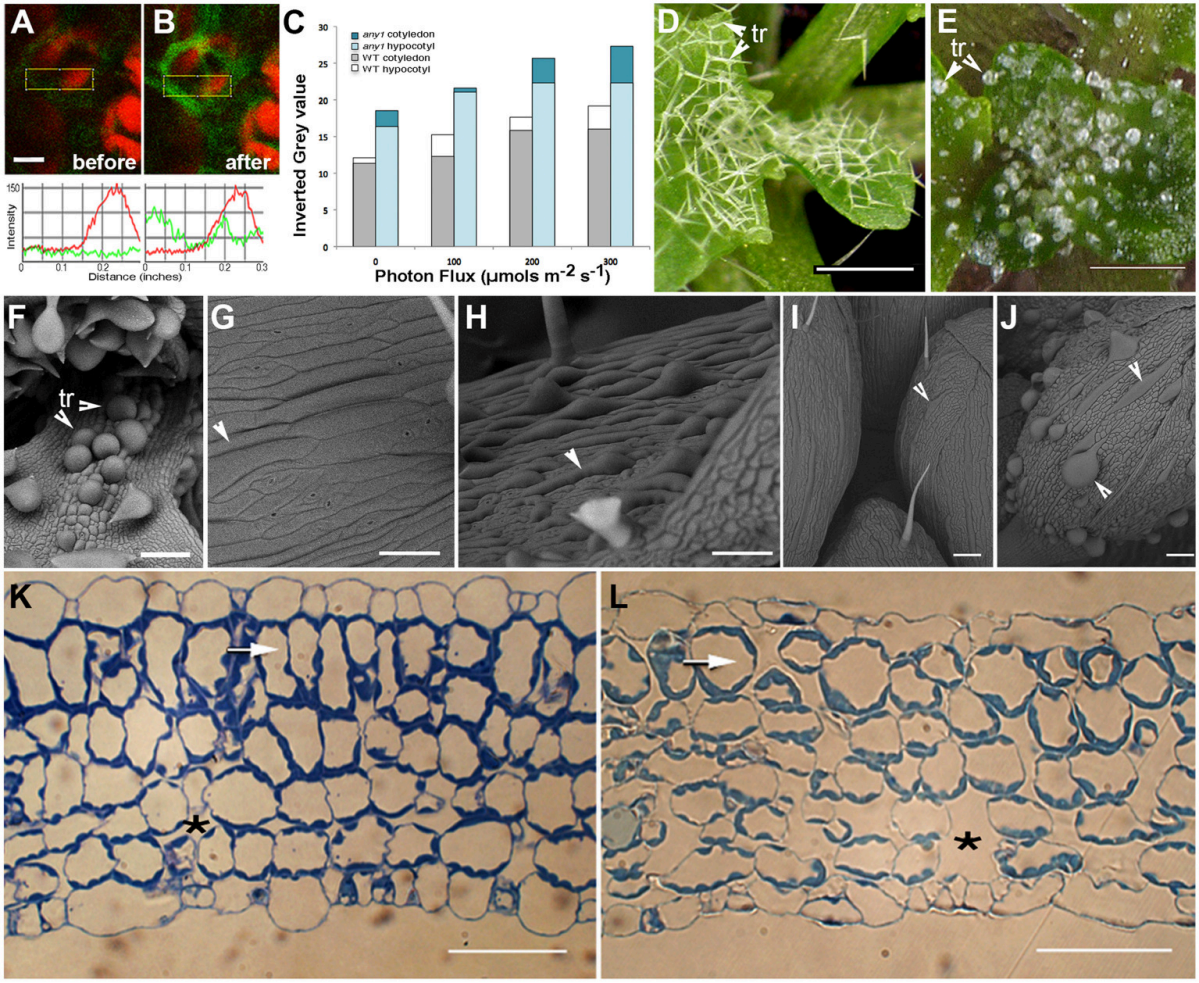
**Observations suggesting changes in subcellular H_**2**_O_**2**_ levels in response to HL and characteristic features of the ***any1*** mutant in comparison with wild type Arabidopsis**. **(A,B)** A leaf epidermal cell in a transgenic *Arabidopsis* plant expressing HyPer-cytosolic probe **(A)** before and **(B)** after exposure to HL. Line traces based on the boxed areas are provided below the fluorescent images and show a nearly 2.5-fold general increase in green fluorescence intensity suggesting an increase in cellular H_2_O_2_ levels. Red chlorophyll auto-fluorescence is from chloroplasts in the mesophyll layer. **(C)** DAB staining of WT and *any1* mutant leaves exposed to increasing light intensity suggesting relatively higher H_2_O_2_ production and catalase activity in *any1* cells. **(D,E)** Leaf epidermal trichomes (tr) in WT **(D)** and the *any1* mutant **(E)** exhibit spectacular differences in growth anisotropy. **(F)** A scanning electron (SEM) image shows that the glassy-appearing trichomes as well as some non-trichome cells near the leaf base appear swollen due to isotropic growth in *any1*. **(G,H)** Arrowheads pointing to elongated cells in WT leaves suggest their relatively flat nature **(G)** as compared to the bulged cells in petioles of *any1* **(H)** mutant. **(I,J)** Giant cells in the sepal epidermis in WT (**I**; arrowhead) are relatively thin and unobtrusive compared to the large, swollen cells in the mutant (**J**; arrowheads). **(K,L)** Toluidine blue-O stained sections of WT **(K)** and *any1* **(L)** leaves at a similar stage of development show differences in the relative size of cells and intercellular spaces (^*^). The sub-epidermal layer in the mutant (arrows) is relatively disorganized and cells show approximately 35% decrease in elongation (19.3 μm vs. 29.7 μm long in wild type). Size bars in **(A)** = 5; **(D,E)** = 250; **(F–J)** = 100; **(K,L)** = 50 μm

It has been reported that the number of peroxisomes producing peroxules increases following 30–45 s of UV irradiation or exposure to H_2_O_2_ (Sinclair et al., 2009). HL is known to result in increased cytosolic ROS (Foyer and Noctor, 2003; Apel and Hirt, 2004). We used two different methods to assess this. First, transgenic Arabidopsis plants expressing the hydrogen peroxide (H_2_O_2_) responsive HyPer-cytosolic probe (Costa et al., 2010) showed a clear spike in fluorescence intensity relative to the basal pre-exposure levels following short exposures of 1–5 min to HL of 850 ± 50 μmol m^−2^ s^−1^. The green fluorescence that had spiked to almost 2.5 times decayed back to basal levels within 8 s of illumination suggesting that the increase in the sub-cellular levels of H_2_O_2_ was transient and fell below fluorescence detection levels quickly (Figures 2A,B). Unless stated otherwise the 4 min exposure time was maintained as the minimum in subsequent experiments.

An alternative approach used the DAB staining method used to assess increased H_2_O_2_ production in tissues over a relatively longer period (Figure 2C). Based on the idea that the plant cell wall acts as micro lens and has a regulatory role in determining the intensity and property of light that reaches the cell interior (Haberlandt, 1914; Vogelmann, 1993; Vogelmann et al., 1996) we used *anisotropy1*, an Arabidopsis mutant for comparison with the wild type (WT) plants. The *any1* mutant in an Arabidopsis cellulose synthase (CesA) gene has reduced cell wall crystallinity (Fujita et al., 2013). As a result, epidermal cells in *any1* are nacreous and in comparison to the WT (Figures 2D,G,I) display increased curvature (Figures 2E,F,H,J) and larger inter-cellular spaces (Figure 2K vs. Figure 2L). Exposing 8 day old seedlings of WT and *any1* that had been kept in the dark for 24 h to achieve basal ROS level to 164 μmol m^−2^ s^−1^ light for 30, 60, and 120 min was followed by DAB staining for the presence of H_2_O_2_. The intensity of staining was higher in *any1* for every treatment (Figure 2C) suggesting that as compared to the WT the mutant plants had higher H_2_O_2_ levels.

Thus, the HL treatment on HyPer-cytosolic plants (Figures 2A,B) provided us with a short time window that could be used for assessing the effects of ROS on peroxisomes and mitochondria while the longer light treatment and DAB staining (Figure 2C) suggested *any1* as potential experimental material that might provide differences in organelle behavior during HL-induced ROS stress. WT and *any1* plants expressing YFP-PTS1 and mitoGFP that highlights peroxisomes (Mathur et al., 2002) and mitochondria, respectively (Logan and Leaver, 2000) were assessed next.

### High light induces elongated peroxules but results in smaller mitochondria

**Figure 3:**
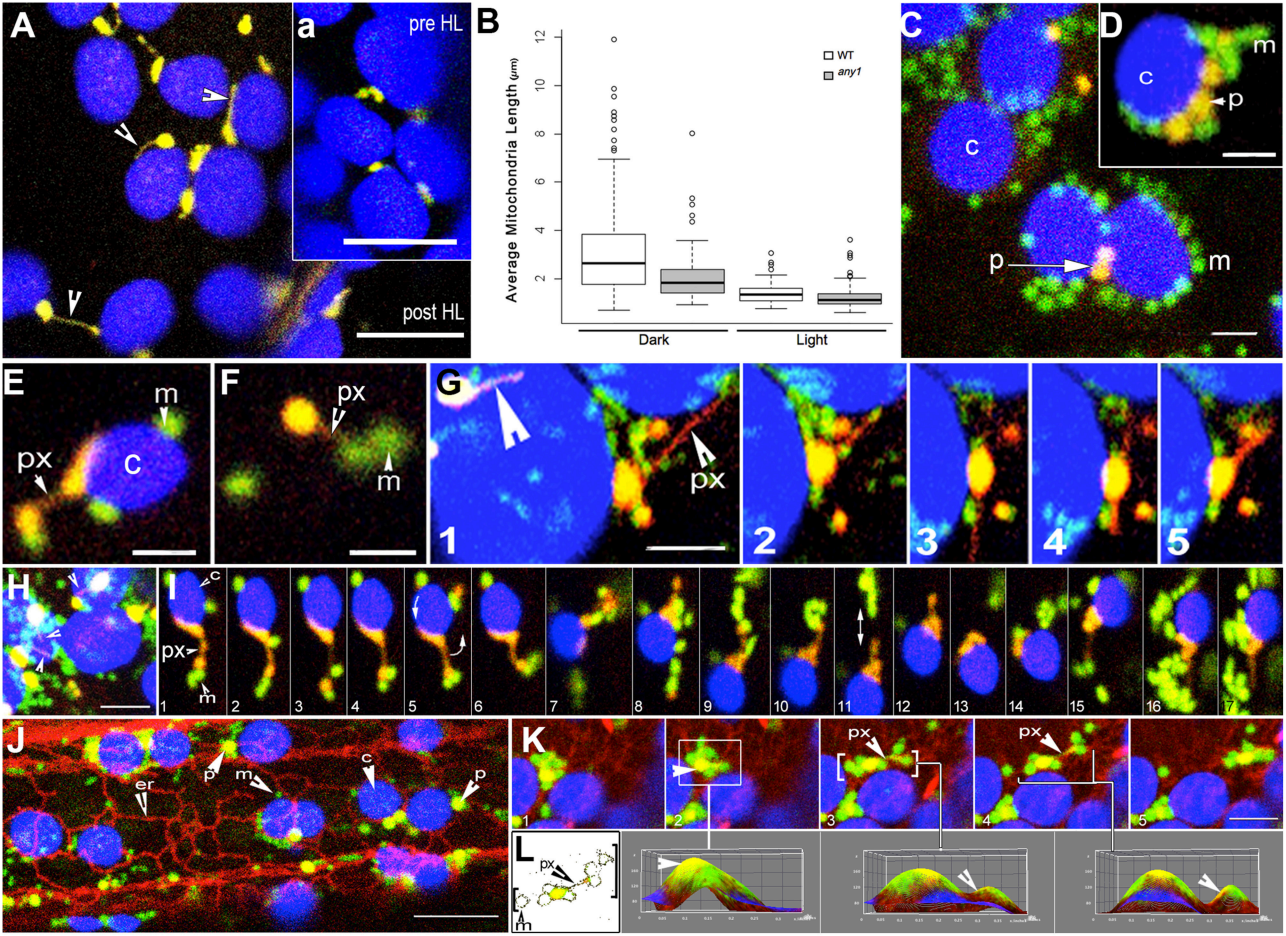
**Peroxisome and mitochondria in wild type and ***any1*** mutant ***Arabidopsis*** plants. (A)** A single cotyledon mesophyll cell exhibits peroxules in the YFP-PTS1 line in the *any1* mutant. Time lapse sequence shown as Movie S4. The inset “**a**” shows a portion of the same cell before HL exposure. **(B)** Mitochondrial length, taken as an indicator of increased cellular ROS is significantly smaller in the *any1* mutant in comparison to WT under both light and dark growth conditions on medium without sugar (*p* < 0.05). **(C)** Mitochondria and peroxisomes often cluster around chloroplasts in cotyledon and hypocotyl cells of WT seedlings following HL exposure (Movie S1 vs. Movie S2). **(D)** Similar clusters to “**c**” are observed in swollen cells of *any1* even under 125 ± 10 μmol m^2^ s^1^ light intensity. Peroxisome numbers are often lower than mitochondria in these clusters. **(E)** A peroxule (px) extended by a chloroplast-associated peroxisome shows mitochondrial (m) association. **(F)** An independent peroxisome with extended peroxule associates with two mitochondria following a 4-min exposure to HL. **(G)** Dynamic extension (frames 1–3), retraction (frame 4) and change of direction of extension (frame 4,5) of a peroxule (px) from a chloroplast associated peroxisome. Multidirectional peroxule extension while the parent peroxisome remains in one location allows interactions with several mitochondria on different sides. A second peroxisome with an extended peroxule is indicated (large arrowhead). **(H)** Single cotyledon cell in the *any1* mutant stably expressing YFP-PTS1 and mitoGFP shows several chloroplast-associated yellow-orange peroxisomes and peroxules (arrowheads) with clusters of mitochondria (green) (Movie S5). **(I)** A series of 17 sequential time-lapse snapshots of a hypocotyl cell of the double transgenic *any1* mutant show a chloroplast-associated peroxisome (orange-yellow) extending a peroxule. Note that the peroxisome-chloroplast association remains strong despite the rotation of the chloroplast (arrow in panel 5) while the mitochondrial population (green) that comes in contact with the extended peroxule is maintained even though individual mitochondria may join and leave the cluster (Movie S6). **(J)** Hypocotyl cell in a triple transgenic Arabidopsis plant shows the typical associations formed between chloroplasts (c-blue) mitochondria (m-green) peroxisomes (p-yellow) and the ER (er-red) following a 4-min HL exposure (Movie S7). **(K,L)** Analysis of sequential snapshots of a mitochondria-peroxisome cluster (yellow-green) moving past chloroplasts (blue) on an ER defined path. 3D surface plots corresponding to panels K2, K3, K4 show clear yellow peaks indicating the presence of peroxisomes within the cluster whereas color thresh-holding and skeletonizing in “L” reveals the presence of a thin peroxule amongst mitochondria in panels K3 and K4. Size bars = **(A,a)** = 5 μm; **(C,D)** = 2.5 μm **(E–I,K)** = 5 μm; **(J)** = 10 μm.

Peroxisomes in both WT and *any1* plants moved as part of the cytoplasmic stream at variable velocities ranging from 1.5 to 7 μm s^−1^ which is consistent with peroxisome motility rates observed earlier (Jedd and Chua, 2002; Mathur et al., 2002). No undue clustering of peroxisomes was observed in *any1* cells prior to HL exposure and attested to their healthy state. However, there was large variability between WT and *any1* cells in terms of peroxisomal response to HL. Only 6 ± 2% peroxisomes in cotyledon epidermal cells (*n* = 50 cells) from 11 to 13 day old WT plants exhibited peroxules after 8–10 min of HL exposure. Comparable cells in *any1*-YFP-PTS1 plants showed 30 ± 7% (*n* = 80 cells) peroxules upon exposure for less than 8 min (Figure 3A,a; Movie S4). Longer exposure time of up to 15 min was sometimes needed for hypocotyl tissue in both plant types, especially if the plants had already been in light for several hours. However, we did not observe chlorophyll bleaching or peroxisome clustering under HL light irradiation conditions in these plants suggesting that the cells, although stressed, were still functional after the prolonged exposure. While the dynamic behavior of peroxules (Movie S4), made it difficult to measure their precise dimensions, cotyledon cells of *any1* often had longer peroxules than similar cells in the WT plants. In addition, as judged by 488 nm laser induced photo-bleaching, the peroxules in *any1* appeared more robust and retained their fluorescence for a longer time as compared to peroxules in wild type plants (data not shown).

Similar HL treatments were carried out on WT and *any1* plants expressing mitoGFP and a comparison of mitochondrial size between the two kinds of plants under dark and light conditions showed significantly smaller mitochondria in the mutant (Figure 3B; Figure S1). Upon exposure to HL, mitochondria in *any1* appeared relatively fuzzy compared to mitochondria in WT cells. Further, the smallest mitochondrial size distribution of 0.5 ± 0.2 μm was observed in large, swollen epidermal cells in *any1* cotyledons whereas WT cotyledon cells maintained a predominant mitochondrial population of ca. 0.8 μm length. Whereas, we have reported earlier that mitochondria in WT plants grown in the dark appear long and tubular and undergo rapid fission upon exposure to light (Jaipargas et al., 2015), we were unable to ascertain a clear size difference between mitochondria in cotyledons of dark grown and light exposed *any1* plants.

Having made our baseline observations for both WT and *any1* plants under HL stress we investigated the behavior of peroxisomes and mitochondria simultaneously in double transgenic plants.

### Small mitochondria cluster around peroxules

Exposing 7–10 day old double transgenic, non-mutant plants expressing YFP-PTS1 and mitoGFP to HL for 4 min resulted in altered interactions between organelles. Whereas, the majority of peroxisomes and mitochondria moved independently before HL irradiation there were clear clusters of organelles after the treatment (Figure 3C). However, the degree of organelle clustering varied widely between different cells and therefore cannot be provided as an average number. Mitochondria and peroxisomes of nearly similar sizes appeared together more often after HL irradiation while major clustering took place around chloroplasts. Notably, the peroxisome-mitochondria clusters around chloroplasts did not contain an equal number of the two organelles. In 72% of the clusters observed (*n* = 100) the ratio of mitochondria to peroxisomes was nearly double while extreme ratios approaching 5:1 were also observed (Figures 3C,D). The clustering appeared to take place through changes in the speed of cytoplasmic streaming and a perceptible slowdown occurred over 20 min of imaging. As reported earlier (Jaipargas et al., 2015), imaging of the same cell for more than 15 min using the 488 nm laser led to significant chlorophyll photo-bleaching and a hypoxia induced swelling of mitochondria. While mitochondria-peroxisome clusters observed in the non-mutant background exhibited peroxules only sporadically, cotyledon cells in *any1* readily exhibited a high frequency of these extensions. In both cases the dynamic, extending-retracting peroxules had clusters of small mitochondria with diameters of 0.7 ± 0.2 μm around them (Figures 3E–I). Whereas, mitochondria exhibit a wide morphological range from tubular to flattened disc-shaped giant mitochondria (Cavers, 1914; Lewis and Lewis, 1914; Van Gestel and Verbelen, 2002; Schrader, 2006; Logan, 2010; Jaipargas et al., 2015), we observed only the smallest forms clustering around peroxules. A cluster was scored as sustained if it was maintained for a minimum of five frames or 20 s (e.g., Figure 3I). Although such clustering around peroxules was observed in 65 independent time-lapse imaging sequences the fact that some mitochondria joined the cluster while others left it (Figures 3G,I; Movie S3, 5, 6) did not allow us to ascertain the time that a single mitochondrion might spend in possible interaction with the peroxule. In addition several clusters were maintained even though the peroxule extended and retracted or the peroxisome swiveled around while moving as part of the cytoplasmic stream (Figure 3I; Movie S6). The independent streaming of mitochondria past different peroxules without joining a cluster suggested that only a particular subset of the mitochondrial population becomes involved in the sustained interaction.

A possible mechanism for organelle clustering in response to HL was investigated next.

### The endoplasmic reticulum (ER) enmeshes peroxisomes, mitochondria, and chloroplasts

Along with specific and highly conserved mitochondrial fission factors (Schrader, 2006) the ER has been shown to mediate mitochondrial fission in yeasts, animal cells, and plants (Friedman et al., 2011; Jaipargas et al., 2015). The resultant mitochondria fall into the size range represented in the mitochondrial cluster around peroxules. A loose ER cage has been described around chloroplasts (Schattat et al., 2011) and it has been shown that under HL the ER accumulates around chloroplasts to form an ER-chloroplast nexus (Griffing, 2011). Moreover, earlier studies have implicated the ER in peroxisome behavior and peroxule extension (Sinclair et al., 2009; Barton et al., 2014). As the ER appears to be a major contributor to organelle motility, pleomorphy, and fission, we investigated its rearrangement as a possible mechanism for the observed clustering of peroxisomes, mitochondria and chloroplasts.

Triple transgenic lines expressing YFP-PTS1, mitoGFP, and RFP-ER were created. Using chlorophyll auto-fluorescence to distinguish chloroplasts allowed simultaneous visualization of all four organelles (Figure 3J). In more than 60% of observations (180 frames from seven time-lapse sequences) mitochondria and peroxisomes in the vicinity of chloroplasts were drawn into an ER-chloroplast nexus following HL exposure. Use of ImageJ color thresholding and 3D surface plot functions showed that peroxisomes and peroxules existed within the clusters (Figures 3K,L). In each case the movement, extension and retraction of peroxules and associated mitochondria occurred in tandem with the dynamic reorganization of neighboring ER tubules (Figure 3K). However, the ER-chloroplast nexus reorganized into dynamic tubules a few minutes after the HL exposure, while the mitochondria-peroxisome-chloroplast association was maintained for much longer periods. This suggested that while reorganization of the ER upon exposure to HL might facilitate increased proximity between organelles, their subsequent aggregation after the ER has reverted to its normal organization may involve other membrane factor(s) that are common to and shared between the organelles.

## Discussion

Live imaging has revealed numerous instances where transient changes in organelle morphology are observed in response to cellular stress (Mathur et al., 2012). For instance, it has been suggested that chloroplasts extend stroma filled tubules called stromules in response to internal ROS accumulation following inhibition of the electron transport chain (pETC; Brunkard et al., 2015) and other plastids respond to increased sugar levels in a cell (Schattat and Klösgen, 2011). Similarly, the rapid fission of tubular mitochondria increases their population in a cell in response to high cytosolic sugar content, high ROS levels and light stimuli (Yoshinaga et al., 2005; Yu et al., 2006, 2008; Jhun et al., 2013; Jaipargas et al., 2015). Here we have shown that HL also induces peroxule formation from peroxisomes. In an earlier study peroxule formation was observed as a direct morphological consequence of increased H_2_O_2_ levels in a plant cell (Sinclair et al., 2009).

The ROS-stress based mechanism for peroxule extension (Sinclair et al., 2009) is reinforced further by the observations presented here on the Arabidopsis *any1* mutant. In comparison to the WT Arabidopsis epidermal cells, the mutant cells have increased curvature due to reduced cellulose crystallinity (Fujita et al., 2013). In general, epidermal cells in plants are known to act as lenses that, depending upon characteristics such as the cell surface area, cell wall composition and the degree and uniformity of curvature, filter, and refract the sunlight that reaches the mesophyll and other internal layers in a plant (Haberlandt, 1914; Vogelmann, 1993; Vogelmann et al., 1996). If the radius of curvature (r), is small, the light is focused with minimal scattering to the top of the mesophyll layer. Conversely, if the radius is large (i.e., the cell is relatively flat), the light gets focussed farther below the epidermis. However, the deeper the focal point of light, the greater is the scattering and hindrance of absorption (Vogelmann et al., 1996). It would therefore be expected that the chloroplasts would be exposed to a higher light intensity if the cells in the outermost epidermal layer are more spherical. One of the consequences of increased light absorbance by the chloroplasts is an increase in the levels of photorespiration associated subcellular ROS. Indeed, as observed by us the typically round and bulbous epidermal cells in *any1* exhibit higher H_2_O_2_ levels as compared to the WT. Expectedly this leads to the increased peroxule formation in *any1* after HL exposure.

Observations on peroxules immediately prompt questions about the source of membranes that must become available for such extensions to be formed. A general mechanism that would involve different peroxisomal membrane proteins (PMPs) including PEX11 isoforms can be suggested. Indeed, the overexpression of different PEX11 isoforms over several hours has been shown to increase peroxisome tubulation and clustering (Delille et al., 2010; Koch et al., 2010). However, as shown here peroxules become visible within less than 5 min of HL-induced ROS stress. Their quick appearance seems to preclude the longer process requiring recruitment and incorporation of different PMPs and fission factors for peroxisome proliferation. Their rapid formation suggests the availability of a source of compatible membranes from the surroundings.

Recent research involving mitochondria derived vesicles (MDVs) suggests interesting possibilities involving mitochondria and peroxisomes (Neuspiel et al., 2008; Andrade-Navarro et al., 2009; Braschi et al., 2010; Mohanty and McBride, 2013). Schematic depictions suggest that transfer of mitochondrial matrix and inter-membrane metabolites or proteins might involve mitochondrial extensions that can increase both surface area and/or physical contact with peroxisomes (Figure 1 in Schumann and Subramani, 2008). Whereas, our observations have not shown any mitochondria extensions that might validate the diagrammatic depictions of Schumann and Subramani (2008) our double transgenics do show clear peroxules that are surrounded by mitochondrial clusters.

Our observations thus strongly suggest that peroxules might act as platforms where mitochondrial outer membrane proteins might become incorporated into an existing peroxisome membrane. Whereas, we have not demonstrated the actual transfer of MDVs and their contents to peroxisomes in living cells, we have often observed the rapid thickening and elongation of peroxules that suggests such a possibility. Notably in all our experiments both mitochondria and peroxisomes are motile before HL exposure and do not appear to interact with each other beyond an occasional coincidental interaction that lasts for 1 or 2 time-lapse frames (about 6 s). Following HL exposure, however, there is a perceptible increase in mitochondria-peroxisome clusters both near and away from chloroplasts. In plants, both mitochondria and peroxisomes are linked metabolically with chloroplasts (Douce et al., 2001; Foyer and Noctor, 2003; Hayashi and Nishimura, 2003; Apel and Hirt, 2004; Ježek and Plecitá-Hlavatá, 2009). The same is true for the ER membranes that surround the different organelles. Since a short random diffusion distance of 1 μm is attributed to H_2_O_2_ (Halliwell and Gutteridge, 1989) all of these organelles might be expected to respond to it. One of the responses to increased ROS is the swelling of ER tubules (Margittai et al., 2008). This could lead to a more crowded ER with reduced flow characteristics that might account for the increased proximity of organelles that become enmeshed in it following HL-exposure.

However, as observed by us the change in ER dynamics and organization around chloroplasts was transient and the ER resumed its dynamic behavior after a few minutes. However, mitochondria and peroxisomes remained clustered around chloroplasts for a much longer period. We speculated that the 3-organelle cluster is maintained due to the presence of one or more proteins that is/are shared between them. FISSION1 represents such a protein. This tail-anchored membrane protein is highly conserved between different eukaryotes, exists with the same membrane topology in both peroxisomal and mitochondrial membranes, and has been shown to help recruit the DRPs to the peroxisomal and mitochondrial membrane for their eventual fission (Schumann and Subramani, 2008). Most important, in plants FIS1A has been shown to localize to peroxisomes, mitochondria and chloroplasts (Ruberti et al., 2014).

**Figure 4:**
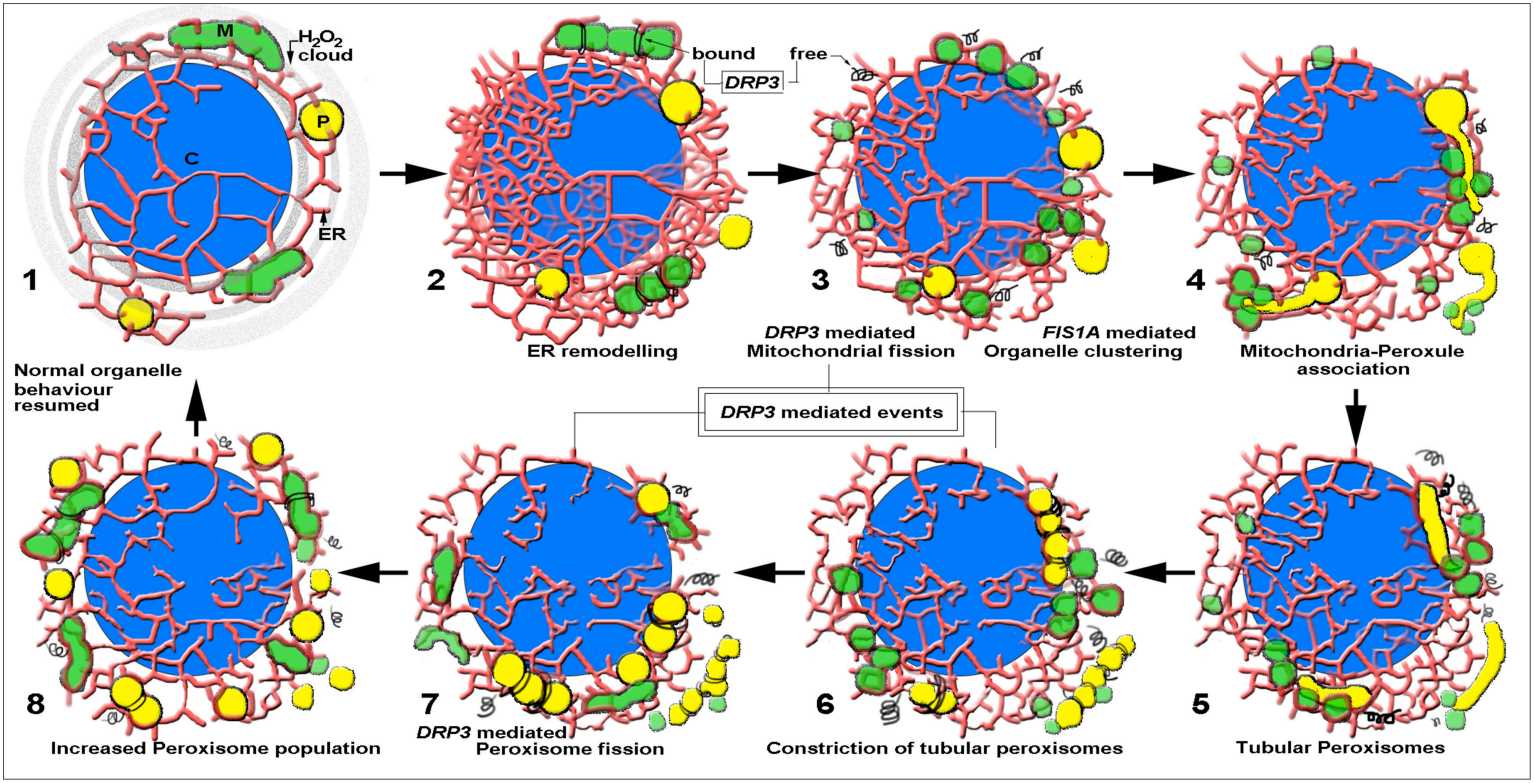
**A speculative diagrammatic depiction of the sequence of responses and interactions involving chloroplasts (C), endoplasmic reticulum (ER), mitochondria (M) and peroxisomes (P) following transient high-light (HL) induced ROS stress**. 1. A chloroplast acts as a focal point for H_2_O_2_ release into the cell. Neighboring membranes and organelles are affected before the H_2_O_2_-cloud dissipates and gets neutralized. 2. ER polygons and cisternae around a chloroplast are affected by the transient increase in H_2_O_2_ and organize into an ER-chloroplast nexus that brings peroxisomes and mitochondria closer to the chloroplast. Mitochondria undergo fission rapidly in a DRP3 (shown as spirals) aided manner in response to increased H_2_O_2_/ROS. 3. The ER-chloroplast nexus disassembles but FIS1A present on chloroplast, mitochondrial and peroxisomal membranes tethers the organelles together and promotes their interactions. Note that the cytosolic concentration of free-DRP3 is speculated to increase after mitochondrial fission. 4. Small mitochondria cluster around thin dynamic peroxules extended by spherical peroxisomes. The sustained proximity might aid both non-vesicular and vesicular interactions between the organelles as well as promote the redistribution of DRP3 proteins. 5. Spherical peroxisomes are molded into tubules of nearly uniform diameter similar to that of tubular mitochondria. 6. Tubular peroxisomes become beaded as DRP3 binds and constricts at regular intervals. 7. Beaded peroxisomes undergo fission at DRP3-constricted sites through ER-aided contortions. 8. The peroxisomal population in the cell increases and aids rapid scavenging of H_2_O_2_/ROS to basal levels. Mitochondria resume their normal ER-mediated fusion-fission behavior. Normalcy is restored within the cell and the increased peroxisomal population fortifies the cell temporarily against harsher ROS stress.

Our observations suggest a simple, presently quite speculative but testable model (Figure 4). As shown by us HL-induces a general increase in cellular ROS through the combined emanations from chloroplasts and other organelles. Increased mitochondrial ROS triggers their extensive fission and results in small, distressed mitochondria whose further breakdown would lead the cell into a cell death pathway. The increased subcellular ROS also has an effect on the ER and increases organelle proximity and interactivity. The close proximity possibly facilitates exchange of membranes proteins perhaps in a MDV mediated manner (Braschi et al., 2010; Mohanty and McBride, 2013). Increased availability of membranes and specific PMPs allows peroxules to attain a particular diameter that facilitates the recruitment of cytosol localized fission proteins such as DRP3A/B that might have been released following mitochondrial fission (Roux et al., 2010; Mears et al., 2011). DRP3 binding and constriction creates tubular-beaded peroxisomes and eventually results in peroxisome proliferation.

While details of this rather simplistic interpretation of HL induced events aimed at combatting ROS stress in a plant cell continue to be assessed critically, the work presented here clearly provides visual proof of sustained interactions between peroxisomes and mitochondria in living plant cells with peroxules acting as transient interaction platforms.

## Author contributions

EJ and JM designed experiments and co-wrote the manuscript. NM and FD provided expert help with experiments and plant materials. GW provided seeds of *any1* mutant. All authors contributed critical comments and corrections and have approved the manuscript.

### Conflict of interest statement

The authors declare that the research was conducted in the absence of any commercial or financial relationships that could be construed as a potential conflict of interest.

## Supplementary material

Figure S1 **A comparison of predominant mitochondrial size between dark grown plants of ***Arabidopsis thaliana*** ecotype Columbia “A” and the ***any1*** mutant “B” expressing GFP targeted to mitochondria**. Size bar applies for **(A,B)** = 5 μm.

Movie S1 **Time-lapse sequence taken over 2 min of a portion of a cell from a low- light (60 μmol m^**−2**^ s^**−1**^) grown double transgenic Arabidopsis seedling showing the general sub-cellular motility and lack of sustained association between chloroplasts (depicted in blue), mitochondria (green), and peroxisomes (yellow)**.

Movie S2 **The close association of mitochondria (green), peroxisomes (yellow) and a single chloroplast in a cell exposed to HL for 4-min and then tracked over approximately 7.5 min**. Note that the association between the 3-peroxisomes on the plastid is maintained throughout while mitochondria appear to have relatively short but sustained contacts with the other two organelles.

Movie S3 **A time-lapse sequence taken over nearly 5.5 min shows mitochondria (green) and their strong but transient association with long peroxules (orange–yellow) extended from a chloroplast-attached peroxisome and an independent peroxisome (top left)**.

Movie S4 **A time-lapse sequence taken over 6 min of a cotyledon cell in the ***any1*** mutant expressing a peroxisomal targeted YFP shows numerous peroxules being extended and retracted in response to HL exposure for 4-min**.

Movie S5 **Portion of a single cotyledon cell from a double transgenic ***any1*** seedling exposed to HL for 4-min exhibits numerous chloroplasts (blue) surrounded by small, punctate mitochondria (green), and peroxisomes (orange–yellow) with the latter extending and retracting 8–15 μm long peroxules**.

Movie S6 **Portion of a cell in an ***any1*** plant double transgenic for YFP-PTS1-highlighted peroxisomes (yellow) and GFP-targeted mitochondria (green) observed over nearly 5 min shows organelle association with a chloroplast (auto-fluorescent chlorophyll depicted in blue)**. A peroxisome becomes closely associated with the chloroplast after a 4-min HL treatment and repeatedly extends and retracts thin, tubular peroxules that interact transiently with mitochondria in their vicinity.

Movie S7 **A portion of a cell from a triple transgenic Arabidopsis plant expressing YFP- PTS1 (peroxisomes-yellow), mitoGFP (green mitochondria) and RFP-ER (red) shows the phenomenon of aggregation around some chloroplasts (blue) after a 4-min HL illumination**.

## References

[B1] Andrade-Navarro M. A. Sanchez-Pulido L. McBride H. M. (2009). Mitochondrial vesicles: an ancient process providing new links to peroxisomes. Curr. Opin. Cell Biol. 21, 560–567. 10.1016/j.ceb.2009.04.005 19423315

[B2] Apel K. Hirt H. (2004). Reactive oxygen species: metabolism, oxidative stress, and signal transduction. Ann. Rev. Plant Biol. 55, 373–399. 10.1146/annurev.arplant.55.031903.141701 15377225

[B3] Arimura S. Aida G. P. Fujimoto M. Nakazono M. Tsutsumi N. (2004). Arabidopsis dynamin-like protein 2a (ADL2a), like ADL2b, is involved in plant mitochondrial division. Plant Cell Physiol. 45, 236–242. 10.1093/pcp/pch024 14988495

[B4] Arimura S. Tsutsumi N. (2002). A dynamin-like protein (ADL2b), rather than FtsZ, is involved in Arabidopsis mitochondrial division. Proc. Natl. Acad. Sci. U.S.A. 99, 5727–5731. 10.1073/pnas.082663299 11960028 PMC122839

[B5] Barton K. A. Jaipargas E. A. Griffiths N. Mathur J. (2014). Live-imaging of peroxisomes and peroxules in plants, in Molecular Machines Involved in Peroxisome Biogenesis and Maintenance, eds Brocard C. Hartig A. (Wien: Springer-Verlag), 233–253. 10.1007/978-3-7091-1788-0_10

[B6] Barton K. Mathur N. Mathur J. (2013). Simultaneous live-imaging of peroxisomes and the ER in plant cells suggests contiguity but no luminal continuity between the two organelles. Front. Physiol. 24:196 10.3389/fphys.2013.00196 PMC3721060 23898304

[B7] Belousov V. V. Fradkov A. F. Lukyanov K. A. Staroverov D. B. Shakhbazov K. S. Terskikh A. V.. (2006). Genetically encoded fluorescent indicator for intracellular hydrogen peroxide. Nat. Methods 3, 281–286. 10.1038/nmeth866 16554833

[B8] Bhattacharjee S. (2011). Sites of generation and physicochemical basis of formation of reactive oxygen species in plant cell, in Reactive Oxygen Species and Antioxidants in Higher Plants, ed Gupta S. D. (Enfield, NH: Science Publishers), 1–30.

[B9] Bleazard W. McCaffery J. M. King E. J. Bale S. Mozdy A. Tieu Q.. (1999). The dynamin-related GTPase Dnm1 regulates mitochondrial fission in yeast. Nat. Cell Biol. 1, 298–304. 10.1038/13014 10559943 PMC3739991

[B10] Braschi E. Goyon V. Zunino R. Mohanty A. Xu L. McBride H. M. (2010). Vps35 mediates vesicle transport between the mitochondria and peroxisomes. Curr. Biol. 20, 1310–1315. 10.1016/j.cub.2010.05.066 20619655

[B11] Brookes P. S. Yoon Y. Robotham J. L. Anders M. W. Sheu S. (2004). Calcium, ATP, and ROS: a mitochondrial love-hate triangle. Am. J. Physiol. 287, C817–C833. 10.1152/ajpcell.00139.2004 15355853

[B12] Brunkard J. O. Runkel A. M. Zambryski P. C. (2015). Chloroplasts extend stromules independently and in response to internal redox signals. Proc. Natl. Acad. Sci. U.S.A. 112, 10044–10049. 10.1073/pnas.1511570112 26150490 PMC4538653

[B13] Cavers F. (1914). Chondriosomes (mitochondria) and their significance. New Phytol. 13, 96–109. 10.1111/j.1469-8137.1914.tb05742.x

[B14] Clough S. J. Bent A. F. (1998). Floral dip: a simplified method for Agrobacterium-mediated transformation of *Arabidopsis thaliana*. Plant J. 16, 735–743. 10.1046/j.1365-313x.1998.00343.x 10069079

[B15] Corpas F. J. (2015). What is the role of hydrogen peroxide in plant peroxisomes? Plant Biol. (Stuttg). 17, 1099–1103. 10.1111/plb.12376 26242708

[B16] Corpas F. J. Barroso J. B. Palma J. M. del Río L. A. (2013). Peroxisomes as cell generators of reactive nitrogen species (RNS) signal molecules. Subcell. Biochem. 69, 283–298. 10.1007/978-94-007-6889-5_15 23821154

[B17] Costa A. Drago I. Behera S. Zottini M. Pizzo P. Schroeder J. I.. (2010). H_2_O_2_ in plant peroxisomes: an *in vivo* analysis uncovers a Ca^(2+)^-dependent scavenging system. Plant J. 62, 760–772. 10.1111/j.1365-313X.2010.04190.x 20230493 PMC2884081

[B18] Delille H. K. Agricola B. Guimaraes S. C. Borta H. Lüers G. H. Fransen M.. (2010). Pex11pβ-mediated growth and division of mammalian peroxisomes follows a maturation pathway. J. Cell Sci. 123, 2750–2762. 10.1242/jcs.062109 20647371

[B19] Douce R. Bourguignon J. Neuburger M. Rebeille F. (2001). The glycine decarboxylase system: a fascinating complex. Trends Plant Sci. 6, 167–176. 10.1016/S1360-1385(01)01892-1 11286922

[B20] Foyer C. H. Noctor G. (2003). Redox sensing and signalling associated with reactive oxygen in chloroplasts, peroxisomes and mitochondria. Physiol. Plant 119, 355–364. 10.1034/j.1399-3054.2003.00223.x

[B21] Friedman J. R. Lackner L. L. West M. DiBenedetto J. R. Nunnari J. Voeltz G. K. (2011). ER tubules mark sites of mitochondrial division. Science 334, 358–362. 10.1126/science.1207385 21885730 PMC3366560

[B22] Fujita M. Himmelspach R. Ward J. Whittington A. Hasenbein N. Liu C.. (2013). The *anisotropy1* D604N mutation in the Arabidopsis Cellulose Synthase1 catalytic domain reduces cell wall crystallinity and the velocity of cellulose synthase complexes. Plant Physiol. 162, 4–85. 10.1104/pp.112.211565 23532584 PMC3641231

[B23] Gechev T. Petrov V. Minkov I. (2010). Reactive oxygen species and programmed cell death in Reactive Oxygen Species and Antioxidants in Higher Plants, ed Gupta D. S. (Enfield, NH: Science Publishers), 65–78.

[B24] Griffing L. R. (2011). Laser stimulation of the chloroplast/endoplasmic reticulum nexus in tobacco transiently produces protein aggregates (boluses) within the endoplasmic reticulum and stimulates local er remodeling. Mol. Plant 4, 886–895. 10.1093/mp/ssr072 21873618

[B25] Haberlandt G. (1914). Physiological Plant Anatomy. London: MacMillan, 613–630.

[B26] Halliwell B. Gutteridge L. M. C. (1989). Free Radicals in Biology and Medicine, 2nd Edn. Oxford: Clarendon.

[B27] Hayashi M. Nishimura M. (2003). Entering a new era of research on plant peroxisomes. Curr. Opin. Plant Biol. 6, 577–582. 10.1016/j.pbi.2003.09.012 14611956

[B28] Hoefnagel M. H. N. Atkin O. K. Wiskich J. T. (1998). Interdependence between chloroplasts and mitochondria in the light and the dark. Biochim. Biophys. Acta 1366, 235–255. 10.1016/S0005-2728(98)00126-1

[B29] Jaipargas E. A. Barton K. A. Mathur N. Mathur J. (2015). Mitochondrial pleomorphy in plant cells is driven by contiguous ER dynamics. Front. Plant Sci. 6:783. 10.3389/fpls.2015.00783 26442089 PMC4585081

[B30] Jedd G. Chua N. H. (2002). Visualization of peroxisomes in living plant cells reveals actomyosin-dependent cytoplasmic streaming and peroxisome budding. Plant Cell Physiol. 43, 384–392. 10.1093/pcp/pcf045 11978866

[B31] Ježek P. Plecitá-Hlavatá L. (2009). Mitochondrial reticulum network dynamics in relation to oxidative stress, redox regulation, and hypoxia. Int. J. Biochem. Cell Biol. 41, 1790–1804. 10.1016/j.biocel.2009.02.014 19703650

[B32] Jhun B. S. Lee H. Jin Z. G. Yoon Y. (2013). Glucose stimulation induces dynamic change of mitochondrial morphology to promote insulin secretion in the insulinoma cell line INS- 1E. PLoS ONE 8:e60810. 10.1371/journal.pone.0060810 23565276 PMC3614983

[B33] Kobayashi S. Tanaka A. Fujiki Y. (2007). Fis1, DLP1, and Pex11p coordinately regulate peroxisome morphogenesis. Exp. Cell Res. 313, 1675–1686. 10.1016/j.yexcr.2007.02.028 17408615

[B34] Koch A. Schneider G. Luers G. H. Schrader M. (2004). Peroxisome elongation and constriction but not fission can occur independently of dynamin-like protein 1. J. Cell Sci. 117, 3995–4006. 10.1242/jcs.01268 15286177

[B35] Koch A. Yoon Y. Bonekamp N. A. McNiven M. A. Schrader M. (2005). A role for Fis1 in both mitochondrial and peroxisomal fission in mammalian cells. Mol. Biol. Cell 16, 5077–5086. 10.1091/mbc.E05-02-0159 16107562 PMC1266408

[B36] Koch J. Kornelija P. Huber A. Ellinger A. Hartig A. Kragler F.. (2010). PEX11 family members are membrane elongation factors that coordinate peroxisome proliferation and maintenance. J. Cell. Sci. 123, 3389–3400. 10.1242/jcs.064907 20826455

[B37] Lewis M. R. Lewis W. H. (1914). Mitochondria (and other cytoplasmic structures) in tissue cultures. Am. J. Anat. 17, 339–401. 10.1002/aja.1000170304

[B38] Li X. Gould S. J. (2003). The dynamin-like GTPase DLP1 is essential for peroxisome division and is recruited to peroxisomes in part by PEX11. J. Biol. Chem. 278, 17012–17020. 10.1074/jbc.M212031200 12618434

[B39] Lingard M. J. Gidda S. K. Bingham S. Rothstein S. J. Mullen R. T. Trelease R. N. (2008). Arabidopsis PEROXIN11c-e, FISSION1b, and DYNAMIN-RELATED PROTEIN3A cooperate in cell cycle-associated replication of peroxisomes. Plant Cell 20, 1567–1585. 10.1105/tpc.107.057679 18539750 PMC2483373

[B40] Lingard M. J. Trelease R. N. (2006). Five Arabidopsis peroxin 11 homologs individually promote peroxisome elongation, duplication, or aggregation. J. Cell Sci. 119, 1961–1972. 10.1242/jcs.02904 16636080

[B41] Logan D. C. (2010). Mitochondrial fusion, division and positioning in plants. Biochem. Soc. Trans. 38, 789–795. 10.1042/BST0380789 20491666

[B42] Logan D. C. Leaver C. J. (2000). Mitochondria-targeted GFP highlights the heterogeneity of mitochondrial shape, size and movement within living plant cells. J. Exp. Bot. 51, 865–871. 10.1093/jexbot/51.346.865 10948212

[B43] Logan D. C. Scott I. Tobin A. K. (2004). ADL2a, like ADL2b, is involved in the control of higher plant mitochondrial morphology. J. Exp. Bot. 55, 783–785. 10.1093/jxb/erh073 14754924

[B44] Mano S. Nakamori C. Kondo M. Hayashi M. Nishimura M. (2004). An Arabidopsis dynamin-related protein, DRP3A, controls both peroxisomal and mitochondrial division. Plant J. 38, 487–498. 10.1111/j.1365-313X.2004.02063.x 15086806

[B45] Margittai E. Löw P. Szarka A. Csala M. Benedetti A. Bánhegyi G. (2008). Intraluminal hydrogen peroxide induces a permeability change of the endoplasmic reticulum membrane. FEBS 582, 4131–4136. 10.1016/j.febslet.2008.11.012 19038256

[B46] Mathur J. Mammone A. Barton K. A. (2012). Organelle extensions in plant cells. J. Int. Plant Biol. 54, 851–867. 10.1111/j.1744-7909.2012.01175.x 23046073

[B47] Mathur J. Mathur N. Hülskamp M. (2002). Simultaneous visualization of peroxisomes and cytoskeletal elements reveals actin and not microtubule-based peroxisome motility in plants. Plant Physiol. 128, 1031–1045. 10.1104/pp.011018 11891258 PMC152215

[B48] Mears J. A. Lackner L. L. Fang S. Ingerman E. Nunnari J. Hinshaw J. E. (2011). Conformational changes in Dnm1 support a contractile mechanism for mitochondrial fission. Nat. Struct. Mol. Biol. 18, 20–26. 10.1038/nsmb.1949 21170049 PMC3059246

[B49] Mohanty A. McBride H. M. (2013). Emerging roles of mitochondria in the evolution, biogenesis, and function of peroxisomes. Front. Physiol. 4:268. 10.3389/fphys.2013.00268 24133452 PMC3783979

[B50] Mozdy A. D. McCaffery J. M. Shaw J. M. (2000). Dnm1p GTPase- mediated mitochondrial fission is a multi-step process requiring the novel integral membrane component Fis1p. J. Cell Biol. 151, 367–380. 10.1083/jcb.151.2.367 11038183 PMC2192649

[B51] Murashige T. Skoog F. (1962). A revised medium for rapid growth and bioassays with tobacco tissue cultures. Physiol. Plant 15, 473–797. 10.1111/j.1399-3054.1962.tb08052.x

[B52] Neuspiel M. Schauss A. C. Braschi E. Zunino R. Rippstein P. Rachubinski R. A.. (2008). Cargo-selected transport from the mitochondria to peroxisomes is mediated by vesicular carriers. Curr. Biol. 18, 102–108. 10.1016/j.cub.2007.12.038 18207745

[B53] Nito K. Kamigaki A. Kondo M. Hayashi M. Nishimura M. (2007). Functional classification of *Arabidopsis* peroxisome biogenesis factors proposed from analyses of knockdown mutants. Plant Cell Physiol. 48, 763–774. 10.1093/pcp/pcm053 17478547

[B54] Orth T. Reumann S. Zhang X. Fan J. Wenzel D. Quan S.. (2007). The PEROXIN11 protein family controls peroxisome proliferation in Arabidopsis. Plant Cell 19, 333–350. 10.1105/tpc.106.045831 17220199 PMC1820951

[B55] Pitts K. R. Yoon Y. Krueger E. W. McNiven M. A. (1999). The dynamin− like protein DLP1 is essential for normal distribution and morphology of the endoplasmic reticulum and mitochondria in mammalian cells. Mol. Biol. Cell 10, 4403–4417. 10.1091/mbc.10.12.4403 10588666 PMC25766

[B56] Roux A. Koster G. Lenz M. Sorre B. Manneville J. B. Nassoy P.. (2010). Membrane curvature controls dynamin polymerization. Proc. Natl. Acad. Sci. U.S.A. 107, 4141–4146. 10.1073/pnas.0913734107 20160074 PMC2840091

[B57] Ruberti C. Costa A. Pedrazzini E. Lo Schiavo F. Zottini M. (2014). FISSION1A, an Arabidopsis tail-anchored protein, is localized to three subcellular compartments. Mol. Plant 7, 1393–1396. 10.1093/mp/ssu027 24658461

[B58] Schattat M. Barton K. Baudisch B. Klösgen R. B. Mathur J. (2011). Plastid stromule branching coincides with contiguous endoplasmic reticulum dynamics. Plant Physiol. 155, 1667–1677. 10.1104/pp.110.170480 21273446 PMC3091094

[B59] Schattat M. H. Klösgen R. B. (2011). Induction of stromule formation by extracellular sucrose and glucose in epidermal leaf tissue of *Arabidopsis thaliana*. BMC Plant Biol. 11:115. 10.1186/1471-2229-11-115 21846357 PMC3167769

[B60] Schrader M. (2006). Shared components of mitochondrial and peroxisomal division. Biochim. Biophys. Acta Mol. Cell Res. 1763, 531–541. 10.1016/j.bbamcr.2006.01.004 16487606

[B61] Schrader M. Castro I. Fahimi H. D. Islinger M. (2014). Peroxisome morphology in pathologies, in Molecular Machines Involved in Peroxisome Biogenesis and Maintenance, eds Brocard C. Hartig A. (Wien: Springer-Verlag), 125–151. 10.1007/978-3-7091-1788-0_7

[B62] Schrader M. Yoon Y. (2007). Mitochondria and peroxisomes: are the “Big Brother” and the “Little Sister” closer than assumed? BioEssays 29, 1105–1114. 10.1002/bies.20659 17935214

[B63] Schumann U. Subramani S. (2008). Special delivery from mitochondria to peroxisomes. Trends Cell Biol. 18, 253–256. 10.1016/j.tcb.2008.04.002 18468897 PMC3697091

[B64] Scott I. Sparkes I. A. Logan D. C. (2007). The missing link: inter-organellar connections in mitochondria and peroxisomes? Trends Plant Sci. 12, 380–381. 10.1016/j.tplants.2007.08.010 17765598

[B65] Scott I. Tobin A. K. Logan D. C. (2006). BIGYIN, an orthologue of human and yeast FIS1 genes functions in the control of mitochondrial size and number in *Arabidopsis thaliana*. J. Exp. Bot. 57, 1275–1280. 10.1093/jxb/erj096 16510519

[B66] Sinclair A. M. Trobacher C. P. Mathur N. Greenwood J. S. Mathur J. (2009). Peroxule extension over ER-defined paths constitutes a rapid subcellular response to hydroxyl stress. Plant J. 59, 231–242. 10.1111/j.1365-313X.2009.03863.x 19292761

[B67] Stojanovski D. Koutsopoulos O. S. Okamoto K. Ryan M. T. (2004). Levels of human Fis1 at the mitochondrial outer membrane regulate mitochondrial morphology. J. Cell Sci. 117, 1201–1210. 10.1242/jcs.01058 14996942

[B68] Thoms S. Erdmann R. (2005). Dynamin-related proteins and Pex11 proteins in peroxisome division and proliferation. FEBS J. 272, 5169–5181. 10.1111/j.1742-4658.2005.04939.x 16218949

[B69] Tieu Q. Nunnari J. (2000). Mdv1p is a WD repeat protein that interacts with the dynamin-related GTPase, Dnm1p, to trigger mitochondrial division. J. Cell Biol. 151, 353–365. 10.1083/jcb.151.2.353 11038182 PMC2192646

[B70] Van Gestel K. Verbelen J. P. (2002). Giant mitochondria are a response to low oxygen pressure in cells of tobacco (*Nicotiana tabacum* L.). J. Exp. Bot. 53, 1215–1218. 10.1093/jexbot/53.371.1215 11971932

[B71] Vogelmann T. C. Bornman J. F. Yates D. J. (1996). Focusing of light by leaf epidermal cells. Physiol. Plantarum 98, 43–56. 10.1111/j.1399-3054.1996.tb00674.x

[B72] Vogelmann T. C. (1993). Plant tissue optics. Ann. Rev. Plant Physiol. Plant Mol. Biol. 44, 231–251. 10.1146/annurev.pp.44.060193.001311

[B73] Yoon Y. Krueger E. W. Oswald B. J. McNiven M. A. (2003). The mitochondrial protein hFis1 regulates mitochondrial fission in mammalian cells through an interaction with the dynamin-like protein DLP1. Mol.Cell Biol. 23, 5409–5420. 10.1128/MCB.23.15.5409-5420.2003 12861026 PMC165727

[B74] Yoshinaga K. Arimura S. Niwa Y. Tsutsumi N. Uchimiya H. Kawai-Yamada M. (2005). Mitochondrial behaviour in the early stages of ROS stress leading to cell death in *Arabidopsis thaliana*. Ann. Bot. 96, 337–342. 10.1093/aob/mci181 15944174 PMC4246881

[B75] Yu T. Robotham J. L. Yoon Y. (2006). Increased production of reactive oxygen species in hyperglycemic conditions requires dynamic change of mitochondrial morphology. Proc. Natl. Acad. Sci. U.S.A. 103, 2653–2658. 10.1073/pnas.0511154103 16477035 PMC1413838

[B76] Yu T. Sheu S. S. Robotham J. L. Yoon Y. (2008). Mitochondrial fission mediates high glucoseinduced cell death through elevated production of reactive oxygen species. Cardiovasc. Res. 79, 341–351. 10.1093/cvr/cvn104 18440987 PMC2646899

[B77] Zhang X. C. Hu J. P. (2008). FISSION1A and FISSION1B proteins mediate the fission of peroxisomes and mitochondria in Arabidopsis. Mol. Plant 1, 1036–1047. 10.1093/mp/ssn056 19825601

[B78] Zhang X. Hu J. (2009). Two small protein families, DYNAMIN-RELATED PROTEIN3 and FISSION1, are required for peroxisome fission in Arabidopsis. Plant J. 57, 146–159. 10.1111/j.1365-313X.2008.03677.x 18785999

