# Supplementary material for: High Light Intensity Leads to Increased Peroxule-Mitochondria Interactions in Plants
Source: Front Cell Dev Biol. 2016 Feb 4;4:6. doi: 10.3389/fcell.2016.00006 (PMC4740372; doi:10.3389/fcell.2016.00006)

Supplementary information.

Figure S1. A comparison of predominant mitochondrial size between dark grown plants of Arabidopsis thaliana ecotype Columbia 'A' and the any1 mutant 'B' expressing GFP targeted to mitochondria. Size bar = 5 μm.

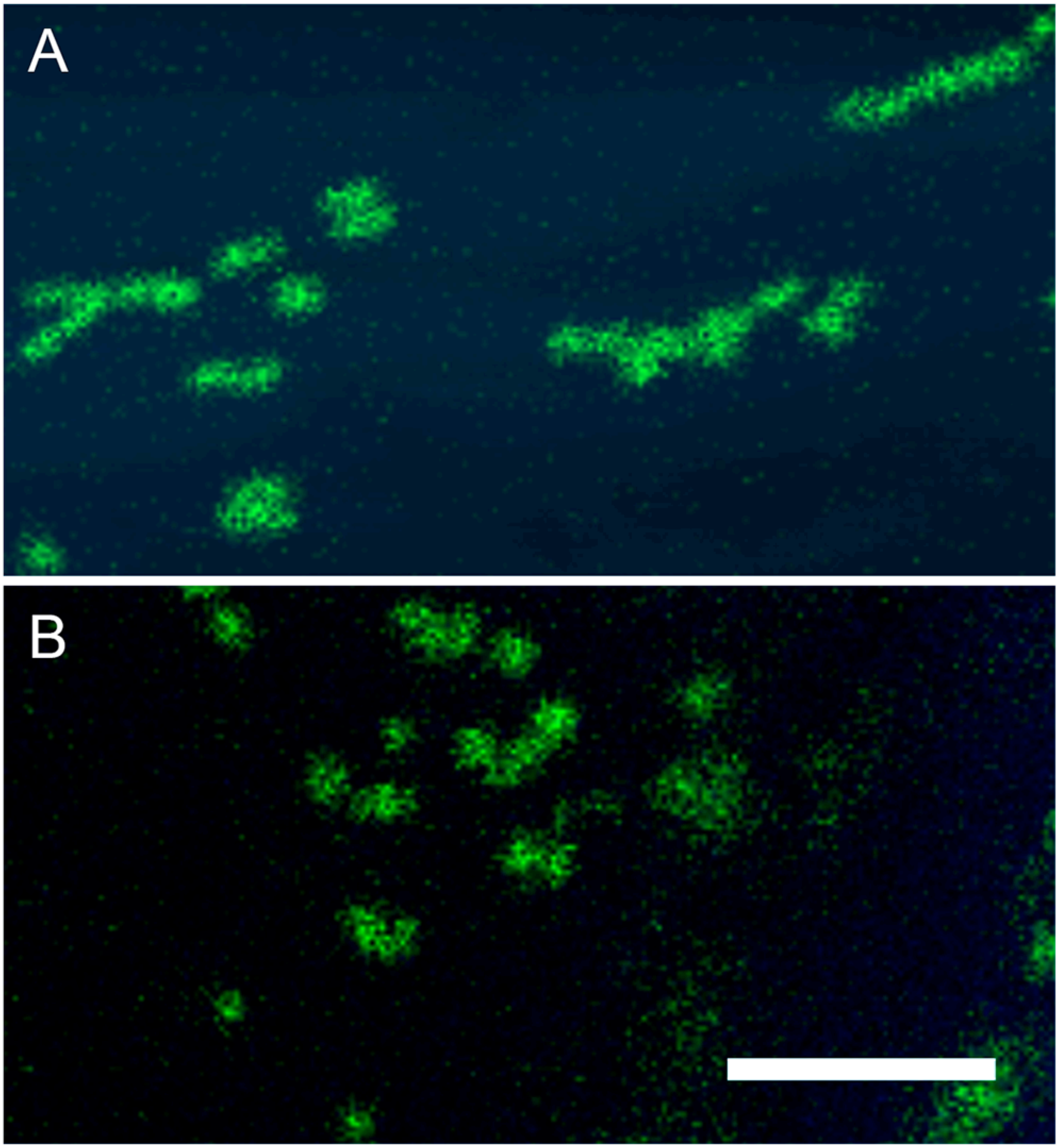

Supplement: Figure S1 — A comparison of predominant mitochondrial size between dark grown plants of Arabidopsis thaliana ecotype Columbia “A” and the any1 mutant “B” expressing GFP targeted to mitochondria. Size bar applies for (A,B) = 5 μm. [file Image1.PDF]
